# Acute Kidney Injury Biomarkers and Hydration Outcomes at the Boston Marathon

**DOI:** 10.3389/fphys.2021.813554

**Published:** 2022-01-03

**Authors:** Whitley C. Atkins, Cory L. Butts, Melani R. Kelly, Chris Troyanos, R. Mark Laursen, Andrew Duckett, Dawn M. Emerson, Megan E. Rosa-Caldwell, Brendon P. McDermott

**Affiliations:** ^1^Exercise Science Research Center, College of Education and Health Professions, University of Arkansas, Fayetteville, AR, United States; ^2^Department of Exercise and Nutrition Sciences, Weber State University, Ogden, UT, United States; ^3^Department of Exercise Science and Pre-Health Professions, Creighton University, Omaha, NE, United States; ^4^Medical Coordinator for the Boston Marathon, Boston, MA, United States; ^5^Sargent College of Health and Rehabilitation Sciences, Boston University, Boston, MA, United States; ^6^Athletic Training Department, Boston University, Boston, MA, United States; ^7^University of Kansas Medical Center, Department of Physical Therapy, Rehabilitation Science, and Athletic Training, Kansas City, KS, United States; ^8^Department of Neurology, Beth Israel Deaconess Medical Center, Harvard Medical School, Boston, MA, United States

**Keywords:** sex-differences, marathon-running, endurance sports, renal stress, dehydration

## Abstract

The purpose of our field study was to investigate the effects of running the Boston Marathon on acute kidney injury (AKI) biomarkers. We hypothesized that biomarker values would be elevated immediately post-marathon but would resolve in the 24-h post-marathon. Secondarily, we sought to identify sex differences related to renal stress. Participants were 65 runners who completed the Boston Marathon (46 ± 9 years, 65.4 ± 10.8 kg). Urine samples were collected at three different time points (pre-marathon, post-marathon, and 24-h post-marathon). Blood samples were collected post-marathon and 24-h post-marathon. Urine specific gravity (USG) and AKI biomarkers were evaluated. Pre-marathon USG (1.012 ± 0.007) was significantly less than post-marathon (1.018 ± 0.008) and 24-h post-marathon (1.020 ± 0.009; *P* < 0.001). Male USG (1.024 ± 0.009) was significantly greater 24-h post-marathon than females (1.017 ± 0.008; *P* = 0.019). Urinary neutrophil gelatinase-associated lipocalin values were significantly greater over time (*P* < 0.001), and there was a main effect of sex with female urinary creatinine (_U_Cr) greater than males at all three time points (*P* = 0.040). Post-marathon_U_Cr (366.24 ± 295.16 mg/dl) was significantly greater than pre-marathon (206.65 ± 145.28.56 mg/dl; *p* < 0.001) and 24-h post-marathon was significantly lower than other time-points (93.90 ± 125.07 mg/dl; *P* < 0.001). Female_U_Cr values were significantly greater than males 24-h post-marathon (*P* < 0.001). There was no difference in serum cystatin C (_S_Cys) values post- or 24-h post-marathon (*P* = 0.178). Serum creatinine (_S_Cr) significantly decreased between post-marathon and 24-h post-marathon, (*P* < 0.001). We can infer that the characteristics unique to the Boston Marathon may have attributed to prolonged elevations in AKI biomarkers. Sex differences were observed during the Boston Marathon warranting further investigation.

## Introduction

In 2018, 1.3 million people completed a marathon road race (Andersen, 2020). Further, more than 300,000 ultramarathons were attempted in 2017 with participation steadily increasing (Scheer, 2019). Distance runners are often viewed as healthy individuals due to their cardiovascular fitness, habitual training volume, and large calorie expenditure (Mansour et al., 2019). However, the prevalence of acute kidney injury (AKI) has been considered as a potential negative consequence of long-distance running but long-term effects are unknown (McCullough et al., 2011; Hewing et al., 2015; Mansour et al., 2017; Knechtle and Nikolaidis, 2018; Poussel et al., 2020; Scheer et al., 2021). AKI is an abrupt decrease in kidney function and can involve structural damage and impairment (Makris and Spanou, 2016). In non-athletes, a single episode of AKI is predictive of future chronic kidney disease (Hoffman and Weiss, 2016). In marathon running, the transient nature of AKI is independent of previous or subsequent participation in marathons (Hoffman and Weiss, 2016). Clinical criterion suggests AKI may affect 40–82% of marathon finishers but resolves within 24 h (McCullough et al., 2011; Mansour et al., 2017). Yet, the clinical criterion for AKI may not be appropriate for marathon runners as the criterion has been developed on clinical populations in resting conditions (Lopes and Jorge, 2013). There still may be long-term consequences of unresolved, or persistent AKI in otherwise healthy distance runners. Given the large amount of participation in marathon running and potential long-term consequences, the need to further investigate the physiological toll of race participation continues to be justified.

The marathon provides an ideal model to study AKI due to a combination of physiological and environmental stressors. The combination of environmental heat stress, exercise dehydration, an elevated core body temperature, decreased renal blood flow, and systemic inflammation is associated with AKI and inherent to many races (Mansour et al., 2017). Others have shown that an increase in mechanical workload (Rojas-Valverde et al., 2019, 2021a,b) or muscle damage increases AKI risk secondary to renal filtration requirements (Hewing et al., 2015). During marathon running, skin blood flow increases while renal blood flow decreases. This reduction in renal blood supply may lead to ischemic tubular damage, especially when combined with heat stress (Mansour et al., 2017; Juett et al., 2020). Further, dehydration, exacerbated in combination with heat stress, may intensify renal stress.

Unique characteristics such as weather, elevation change, or participant finish times between marathons may prolong the resolution of AKI. The Boston Marathon provides an opportunity to investigate these unique characteristics. The race involves a qualification system resulting in a competitive field of runners with faster finish times than other marathons (Breslow et al., 2021). The Boston Marathon course is distinctive in that the first 10 miles are downhill, forcing more eccentric contractions known to elicit muscle damage (Marqueste et al., 2008). The combination of more competitive athletes, faster finish times, warm and humid weather, and downhill running may increase the amount of muscle damage while limiting filtration rate, potentially increasing kidney stress. Our field study served the purpose of investigating the effects of running the Boston Marathon on AKI biomarkers. Our hypothesis was that AKI biomarkers would be elevated immediately post-marathon but would not be resolved in the 24-h post-marathon due to the increased mechanical workload forced by course attributes. Secondarily, we sought to identify sex differences related to renal stress surrounding the Boston Marathon. Participant demographics were 49% female allowing for a comparison between sexes. To our knowledge, sex differences related to AKI have not been reported in previous marathon studies.

## Materials and Methods

### Participants

All registered athletes in the 2019 Boston Athletics Association (B.A.A.) Boston Marathon received an email communication *via* the B.A.A. that described the study and invited their participation. Following initial inquiry from participants, researchers sent a medical screening form. Inclusion criteria required participants to be between the ages of 18–65 years old, either qualified to run the Boston Marathon based on age or ran a marathon in the past 12 months under 4 h. Exclusion criteria included known cardiovascular, respiratory, gastrointestinal, bleeding, inflammatory, metabolic or fluid-electrolyte disorder or other chronic diseases, medication use that could alter renal function, hydration status, gut permeability, or increase risk of exertional heat illness. Additional exclusion criteria included vasovagal syncope as a result of venipuncture. Participants were excluded if they would not be able to attend a 24-h post-marathon data collection session. Seventy-eight registered runners cleared the medical history review and met inclusion criteria. Written informed consent was obtained from all participants prior to the marathon. The study protocol was approved by the University of Arkansas Institutional Review Board.

### Study Design

The day before the race, during athlete check-in at the pre-marathon expo, participants reported to a designated research area to complete a survey regarding demographics, nutrition, medical history, and their intention to take non-steroidal anti-inflammatory drugs (NSAIDs) prior to or during the race. Researchers provided verbal instructions for the procedures of data collection and familiarized participants with the research areas at both the start and finish lines. Researchers were available to answer any questions regarding study procedures for race day. Participants were provided a paper copy of reminders, including the address of the research area and confirmation of appointment time for 24-h post-marathon data collection.

On race day, participants reported to a designated research area, marked with signage, to provide a urine sample. Severe thunderstorms before the race prevented pre-marathon body weight and blood sample collection, and pre-marathon urine and surveys were collected on 55 runners. Immediately after completing the race, participants reported to the designated research area near the finish line. The participants were asked to complete a post-marathon survey and provide urine and blood samples. Sixty-one runners reported to post-marathon data collection. For 24-h post-marathon data collection, participants reported to a research area in a hotel between 3:45AM EST and 12:00PM EST to complete a final survey and provide urine and blood samples. Fifty-two subjects reported to 24-h post-marathon data collection.

### Measurements

#### Environmental Conditions

On race day, dry bulb temperature, wet bulb globe temperature (WBGT), relative humidity (RH), and heat index were recorded every 30-min from 11:00 AM to 4:30 PM near the finish line.

#### Urine Analysis

Urine was collected pre-marathon, post-marathon, and 24-h post-marathon in clear urine specimen collection cups. Urine samples were transported to a local lab where researchers immediately measured and recorded urine specific gravity (USG) *via* refractometry (Master-Sur; Atago, Tokyo, Japan) to assess hydration status (McDermott et al., 2017). The remaining urine was separated into microcentrifuge tubes and frozen. Urine was shipped and stored at −80°C until analyzed. Urine biomarkers used for renal stress assessment included urinary neutrophil gelatinase-associated lipocalin (_U_NGAL) and urinary creatinine (_U_Cr). Urinary biomarkers were quantified according to manufacturer instructions by enzyme-linked immunosorbent assay (ELISA) commercially available kits (R&D Systems, Minneapolis, MN, United States) and colorimetric assay (Cayman Chemical, Ann Arbor, MI, United States).

#### Blood Analysis

Blood samples were collected from cubital veins post-marathon and 24-h post-marathon in serum separator tubes (BD Vacutainer SST, Franklin, NJ, United States) and stored on ice. Blood specimens were transported and centrifuged at a local lab. Once separated, serum was removed and pipetted into microcentrifuge tubes and frozen. Serum specimens were shipped and stored at −80°C until analyzed. Blood biomarkers used for renal stress assessment including serum creatinine (_S_Cr) and serum cystatin C (_S_Cys) were quantified according to manufacturer instructions by ELISA commercially available kits (R&D Systems, Minneapolis, MN, United States) and calorimetric assay (Cayman Chemical, Ann Arbor, MI, United States).

#### Statistical Analysis

All analyses were completed in statistical software, jamovi 1.6.23. Descriptive statistics were used to characterize participant demographics (mean ± standard deviation). Data were tested using a one-way analysis of variance (time). Our time points included pre-marathon, post-marathon, and 24-h post-marathon. To analyze any sex differences, data were tested using a repeated-measures analysis of variance (RM-ANOVA) wherein time (pre-marathon, post-marathon, and 24-h post-marathon) and group (male vs. female) were factors. Huynh–Feldt correction was applied to the omnibus test when the assumption of sphericity was violated. When significant main effect or interactions were identified, *post hoc* comparisons were completed with Holm adjustment due to multiple comparisons. Dependent *t*-tests were used to assess differences in_S_Cys and_S_CR (post-marathon and 24-h post-marathon). Data that failed normality tests (USG and_S_Cys) were analyzed with a Friedman’s test across time and between sexes. Follow-up pairwise analyses were conducted using a Durbin-Conover comparison (USG) and a Wilcoxon signed-rank test (_S_Cys) for individual time point differences. Pearson r bivariate correlations were calculated between renal stress biomarkers. Alpha of 0.05 was set *a priori* to determine significance at the omnibus level.

## Results

### Study Participants

Demographics of study participants are presented in Table 1. Runners finished the marathon with a mean pace of 8:41 (min/mile). A summary of urinalysis and blood biomarkers can be found in Table 2.

**TABLE 1:** Participant demographics.

	Male	Female	Aggregate
Age, year	46 ± 10	45 ± 13	46 ± 10
Sex (*n*)	32	31	63
Weight, kg	73.2 ± 9.7	58.2 ± 6.0*	65.4 ± 10.8
Finishing time, h	3.00 ± 0.45	4.10 ± 0.52*	3.78 ± 0.55

*Denotes significance at p < 0.001 between males and females.

**TABLE 2:** Urinalysis and biomarkers.

	Pre-marathon			Post-marathon			24-h post-marathon		
	Male	Female	Aggregate	Male	Female	Aggregate	Male	Female	Aggregate
USG	1.013 ± 0.008	1.011 ± 0.006	1.012 ± 0.007	1.016 ± 0.007	1.020 ± 0.009^♢^	1.018 ± 0.008*	1.024 ± 0.009	1.017 ± 0.008^♢^	1.020 ± 0.009*
_U_NGAL (ng/mL)	5.48 ± 6.44	17.91 ± 22.09^♢^	12.24 ± 17.83	26.06 ± 26.26	40.16 ± 35.45	33.58 ± 31.95*	59.05 ± 20.16	60.19 ± 18.17	59.70 ± 18.83^+^
_U_Cr (mg/dL)	162.73 ± 154.68	235.70 ± 130.31	201.65 ± 145.28	287.20 ± 251.86	445.28 ± 319.16	366.24 ± 295.16*	44.21 ± 68.97	137 ± 146.83^♢^	93.90 ± 125.07^+^
_S_Cys (mg/dL)	NA			0.14 ± 0.05	0.13 ± 0.03	0.14 ± 0.04	0.14 ± 0.03	0.12 ± 0.03	0.13 ± 0.04
_S_Cr (mg/dL)	NA			2.58 ± 0.67	2.57 ± 0.73	2.58 ± 0.69	2.45 ± 0.71	2.27 ± 0.63	2.37 ± 0.67^+^

*All values are given as mean ± standard deviation.*

*_U_NGAL, urinary neutrophil gelatinase-associated lipocalin;_S_Cys, serum cystatin C;_S_Cr, serum creatinine;_U_Cr, urinary creatinine.*

*Denotes significance at p < 0.001 from pre-marathon.

*^+^Denotes significance at p < 0.001 from post-marathon.*

*^♢^Denotes significance at p < 0.05 from male values.*

### Environmental Data

Environmental data collected at Copley Square, the marathon finish location, showed variable conditions with a WBGT mean of 18.37°C. At the start of the race, WBGT = 16.1°C and at the hottest part of the day (1:43PM) WBGT was 22.1°C with RH of 51.2%. RH throughout the day averaged = 66.5%.

### Urinalysis

Urine specific gravity was significantly affected by time (_$\chi_{2}^{2}$_ = 24.58, *P* < 0.001). Pre-marathon USG was significantly less than post-marathon (*P* < 0.001) and 24-h post-marathon (*P* < 001). There was no significant difference between post-marathon and 24-h post-marathon USG (*P* = 0.421). At pre-marathon data collection, 17.5% of participants were classified as hypohydrated (USG > 1.020) compared to 38.7% post-marathon and 43.9% 24-h post-marathon.

### Renal Stress Biomarkers

Urinary neutrophil gelatinase-associated lipocalin values were significantly elevated post-marathon (*t*_39_ = −7.008, *P* < 0.001) and remained elevated at 24-h post-marathon (*t*_39_ = −11.25, *P* < 0.001)._U_Cr values post-marathon were significantly higher than pre-marathon (*t*_36_ = −4.988, *P* < 0.001), but 24-h post-marathon values were significantly lower than pre-marathon (*t*_36_ = 6.605, *P* < 0.001) and post-marathon timepoints (*t*_36_ = 9.192, *P* < 0.001)._S_Cys concentrations did not differ between post-marathon and 24-h post-marathon timepoints (_$\chi_{1}^{2}$_ = 1.815, *P* < 0.178)._S_Cr significantly decreased between post-marathon and 24-h post-marathon (*F*_1,45_ = 13.70, *P* < 0.001, $\eta_{p}^{2}$ = 0.233).

### Sex Differences

There was an interaction effect between time × sex (*F*_1.89,99.89_ = 11.46, *P* = < 0.001, $\eta_{p}^{2}$ = 0.18) for USG. *Post hoc* contrasts determined that there was a significant difference between USG at 24-h post-marathon related to sex. Male USG was significantly greater 24-h post-marathon than females (*t*_53_ = 3.26, *P* = 0.019).

Urinary neutrophil gelatinase-associated lipocalin data presented a main effect for time (*F*_2.76_ = 81.82, *P* < 0.001, $\eta_{p}^{2}$ = 0.68) and gender (*F*_1,38_ = 4.25 *P* = *0.046*, ^$\eta_{p}^{2}$^ = 0.10). An interaction between sex and time was present (*F*_2,76_ = 3.26, *p* = 0.044), but there was no significant difference between sex at any time point (*P* = 0.067).

Urinary creatinine data showed a main effect for time regardless of sex (*F*_1.50_ = 72.40, *P* < 0.001, $\eta_{p}^{2}$ = 0.67) and sex regardless of time (*F*_1,35_ = 4.532, *P* = 0.040, $\eta_{p}^{2}$ = 0.115). An interaction effect was also present (*F*_1,50_ = 9.57, *P* = 0.001, $\eta_{p}^{2}$ = 0.22). Female_U_Cr values were consistently higher than males with values being significantly greater at 24-h post-marathon (*t*_35_ = 5.482, *P* < 0.001). There was no effect of sex (*Z* = 244.00, *p* = 0.190) on_S_Cys._S_Cr showed a main effect for time (*F*_1.43_ = 14.50, *P* < 0.001, $\eta_{p}^{2}$ = 0.252) but no main effect for sex (*F*_1.43_ = 0.117, *P* < 0.734, $\eta_{p}^{2}$ = 0.003) nor interaction between time × sex (*F*_2.76_ = 81.82, *P* < 0.839, $\eta_{p}^{2}$ = 0.001).

### Biomarker Relationships

Data for correlations between renal stress biomarkers at selected time points are presented in Table 3.

**TABLE 3:** Renal stress biomarker correlations.

Variables	Post-marathon		24-h Post-marathon	
	Pearson r	*p*-value	Pearson r	*p*-value
_S_Cr and_U_NGAL	0.388	0.016*	0.051	0.752
_S_Cr and Cys C	0.362	0.045*	0.208	0.287
_U_NGAL and Cys C	−0.164	0.433	0.082	0.698

*_U_NGAL, urinary neutrophil gelatinase-associated lipocalin;_S_Cys, serum cystatin C;_S_Cr, serum creatinine.*

*Denotes significant correlation (p < 0.05).

## Discussion

This field study investigated the effects of running the Boston Marathon on AKI biomarkers, and as expected, biomarkers of AKI were elevated immediately post-marathon (McCullough et al., 2011; Lippi et al., 2012; Mansour et al., 2017; Juett et al., 2020; Rojas-Valverde et al., 2020). However, renal stress persisted at least 24-h post-marathon. Our primary findings were that (1) short-term renal stress biomarkers remained elevated 24-h post-marathon and (2) some of our participants failed to effectively rehydrate within 24 h of the marathon. Sex differences were evident in (1) USG post-marathon and 24-h post marathon (2),_U_NGAL, and (3)_U_Cr. Our study highlights key differences that exist between physiological outcomes of the runners completing the Boston Marathon compared to previous marathon data (McCullough et al., 2011; Lippi et al., 2012; Mansour et al., 2017).

Our primary biomarker for renal stress was_U_NGAL. Serum elevations of NGAL may be attributed to several sources due to exercise-induced inflammation. Therefore, analysis of NGAL in the urine is a more reliable biomarker to reflect renal impairment (Lippi et al., 2012), especially in the glomerulus and distal tubules. In kidney tubules, NGAL is upregulated after exposure to harmful stimuli including ischemia (Bolignano et al., 2008; Karimzadeh et al., 2017). Our_U_NGAL values for pre- and post-marathon are consistent with previous studies (McCullough et al., 2011; Lippi et al., 2012; Mansour et al., 2017). Previous research identified pre-race_U_NGAL values between 4.4–8.2 ng/mL with elevations to between 35–47 ng/mL post-race (McCullough et al., 2011; Lippi et al., 2012; Mansour et al., 2017). These findings are similar to our baseline mean value of 12.24 ng/mL and post-marathon values of 42 ng/mL. In contrast, previous studies have shown a resolve in_U_NGAL within 24-h post-marathon whereas our data demonstrate a persistent elevation (^+^4.5-fold) in_U_NGAL at 24-h following the race (McCullough et al., 2011; Mansour et al., 2017). To compare, McCullough et al. (2011) and Mansour et al. (2017) found no significant difference between baseline and 24-h post-marathon values and 24-h post-marathon values demonstrated a < 2.3-fold increase compared to baseline. Questions remain regarding the potential consequence of these data, but future research is warranted to determine the time course of renal stress recovery from the Boston Marathon.

Serum cystatin C is produced by all nucleated cells and is filtered by the glomerulus (Mingels et al., 2009). Rather than a biomarker of damage,_S_Cys indicates renal function, with elevated levels denoting impairments (Wołyniec et al., 2020). Several marathon studies have shown that while_S_Cys is elevated post-marathon compared to baseline values, it is typically resolved within 24-h (Mingels et al., 2009; McCullough et al., 2011; Hewing et al., 2015). Unfortunately, we were not able to collect pre-marathon_S_Cys for comparison due to the severe thunderstorms at the start of the race. However, we were able to compare data post-marathon and 24-h post-marathon. Our results differed from multiple studies in that_S_Cys among our participants did not differ between post-marathon and 24-h post-marathon (Mingels et al., 2009; McCullough et al., 2011; Hewing et al., 2015). Our data were in congruence with the findings from Scherr et al. (2011), who found that_S_Cys values were significantly greater from pre-marathon values (mean: 0.077, IQR: 0.071–0.085 mg/dL) at post-marathon (mean: 0.94, IQR:0.86–1.01 mg/dL) and 24-h post-marathon (0.90. IQR 0.81–1.00 mg/dL) and these elevations persisted until 72-h post-marathon (mean: 0.81, IQR: 0.72–0.86 mg/dL) at the 2009 Munich Marathon (Scherr et al., 2011). Persistent elevations of_S_Cys suggest impaired renal function for considerable time frames following difficult courses. These detriments may lead to long-term consequences beyond delayed recovery.

Serum creatinine is a conventional method for measuring AKI (Wołyniec et al., 2020), but because it is a byproduct of creatine, it can be influenced by skeletal muscle activity and muscle size (McCullough et al., 2011),_S_Cr values were significantly lower at 24-h post-marathon compared to immediate post-marathon values. While we are not able to confirm that these values had returned to pre-marathon values, they were trending toward pre-marathon measurements. Previous marathon studies have shown a return to baseline values 24-h post-marathon (Mingels et al., 2009; Hewing et al., 2015). We can infer a resolution from elevated values given additional time but can only speculate on the timing of complete recovery.

Our_U_Cr data are interesting as we do not have a complete explanation of our findings._U_Cr was significantly lower than pre-marathon_U_Cr values at 24-h post-marathon. This decrease suggests a temporary reduction in filtration at the kidney given the increased production of circulating creatinine due to skeletal muscle breakdown. However, a decrease in filtration seems unlikely due to the concomitant decrease in_S_Cr values 24-h post-marathon.

Urine specific gravity was used to reflect acute changes in hydration status in our runners due to our field study design. Although there are limitations in this assessment, USG is known to reflect acute changes in hydration when used within the participant. At the 2015 Hartford Marathon, Mansour et al. (2017) reported no difference in USG from pre-, post-, or 24-h post-marathon. At the 2008 Detroit Marathon, McCullough et al. (2011) reported no significant difference in USG between pre- and 24-h post-marathon. Our data differed in that our pre-marathon USG values were significantly lower than both post-marathon and 24-h post-marathon. While exercise alone may increase USG, our finding suggests that participants may have failed to rehydrate in the hours following the marathon. These data also suggest that hypohydration may have exacerbated or lengthened renal stress due to limitations in filtration following the marathon.

There are several independent factors that may contribute to our findings and explain persistent biomarker elevations reflective of AKI. First, the Boston Marathon course hosts unique features that characterize it differently from previously studied marathons. The disparity between course elevation profiles may provide insight into some of the physiological differences exhibited in marathon competitors. For example, the Hartford Marathon has an elevation gain limited to 608 ft and an elevation loss of 655 ft. The Detroit Marathon is a flat course with a net elevation change of 0 ft. Detroit has one hill that peaks at 100 ft over the course of two miles and one trough of equidistance. Contrarily, the Boston Marathon boasts a gain of 1064 ft and a loss of 1526 ft. The first 10 miles of the marathon are downhill before runners enter the last 16 miles of rolling hills. Eccentric work, specifically downhill running, induces muscle damage and has previously increased elevations in AKI biomarkers (Junglee et al., 2013). Junglee et al. (2013) showed that downhill treadmill running, independent of plasma volume change or nude body mass change, resulted in an increase in USG in a laboratory setting. These authors found a significant difference in_U_NGAL values between downhill running on a treadmill versus running on a treadmill at a 1% gradient (Junglee et al., 2013). In the same study, core temperature was significantly higher with downhill running even when temperature and mean metabolic energy expenditure were matched (Junglee et al., 2013), suggesting that the muscle damage endured from downhill running attributes to a decrease in kidney function. However, hydration status was not well controlled in that study. Regardless, this may explain our findings in that muscle damage would increase USG levels post-marathon due to the excretion of myoglobin from the breakdown of skeletal muscle (Hou et al., 2015).

Further, higher environmental temperatures and concomitant exercise increase the risk for AKI, as blood perfusion to the renal system (and splanchnic organs) is sacrificed in an effort to cool the body (Sawka et al., 2001). Hypohydration exacerbates this as blood volume (plasma) decreases due to sweat losses (Bardis et al., 2013). As plasma volume decreases, runners lose the ability to compensate for the rise in core temperature, perhaps contributing to ischemic tubular kidney injury and cellular kidney damage (Mansour et al., 2017), as well as increased cardiovascular strain and decreased performance (Casa et al., 2010). The temperature at the 2019 Boston Marathon at 1:00 PM EST, 3 h into the marathon, when the majority of participants are still on course, was 20.6°C (63°F). While at most marathons the majority of runners start the race by 7:00 AM and complete the race by late afternoon, thunderstorms postponed the Boston Marathon start and amateur runners did not start the race until 10:00 AM. Therefore, our participants experienced higher temperatures by at least 5.6°C (9°F) than the 2015 Hartford Marathon (temperature at 10:00 AM = 12.2°C, 54°F) and the 2008 Detroit Marathon (temperature at 10 AM = 7.2°C, 45°F) 3 h into each race.

Intensity of exercise is the primary influencer of core temperature in the heat and one unique draw attracting distance runners to the Boston Marathon is that it requires a qualifying time based on age groups for competitors. Therefore, the race typically includes a more seasoned and competitive field. Our data support this notion and demonstrate that our participants represented competitive runners. The average pace of our finishers was 8:41 min/mile, and they finished in a mean of 3.78 h. For comparison, the 2015 Hartford Marathon study included participants with an average finishing time of 4.02 h (Mansour et al., 2017). The 2017 Hartford Marathon and the 2008 Detroit Marathon studies had respective average finish times of 4.24 h (Mansour et al., 2019) and 4.27 h (McCullough et al., 2011). Our participants were 30 s per mile faster than the 2015 Hartford marathon finishers reported by Mansour et al. (2017), and about a minute faster per mile than the participants of the 2017 Hartford Marathon (Mansour et al., 2019) and the 2008 Detroit Marathon (McCullough et al., 2011). Scherr et al. (2011) included participants at the 2009 Munich Marathon with an average finish time of 3.78 h, the same as our participants’ mean finish time. This finding is of particular importance because unlike the findings of Mingels et al. (2009); McCullough et al. (2011), and Hewing et al. (2015),_S_Cys levels did not return to baseline within 24-h after the Munich Marathon or the Boston Marathon. Therefore, intensity (race pace) may be a key contributor of the time needed for the kidneys to recover post-marathon. This assumption is congruent with findings from Siegel and Lopez (Siegel et al., 1980) who found that there was an inverse relationship between finish time and muscle damage in the 1979 Boston Marathon.

We are the first to evaluate sex differences in hydration and renal stress in marathon recovery. Our data suggest that females were unable to rehydrate during the marathon as well as males. Further, renal stress seems more apparent in women, both during and following the marathon. We are uncertain whether or not specific sex-related characteristics or hormones are responsible for our findings. Anthropometrics that may account for some of our results include that female runners are typically smaller in body size and finish times may require females to consistently work at a greater percentage of maximal. However, we believe that further exploration of sex differences in hydration status and AKI in marathon running is warranted to better understand sex-specific physiology during stress and recovery.

We are not able to determine what single factor may have led to differences in AKI biomarkers following the Boston Marathon compared to previous studies. We can assume that it is likely due to a combination of factors. In order to tease out these independent factors (environmental temperature, muscle damage, and exercise intensity), researchers should be in agreement over biomarker assessments for an active population and aim to assess marathons of similar structure (average finish times and course elevations) in similar environmental conditions. The need for multiple data points post-marathon is warranted to assess the time course of recovery as measured by biomarker return to baseline values. Results could further acknowledge best practices for marathon participants to attenuate the risk for AKI or expedite overall recovery. Further, our data from Table 3 suggest that different biomarkers are indicative of a varied time course of recovery. Our data suggest a lack of recovery, but also a lack of correlation demonstrates that_U_NGAL,_S_Cr, and_S_Cys indicate various stressors and are not redundant. This limits our ability to conclude regarding a specific renal stress component or area of the kidney where impairment occurs in response to a marathon.

### Clinical Relevance

Regardless of the mechanisms underlying our findings, it is important for runners to be aware that their renal system is temporarily compromised, and they may require additional recovery time after running the Boston Marathon compared to other marathons. Finishers should prioritize recovery as part of their race plan (McDermott et al., 2017). To facilitate a return to baseline values for euhydration and kidney stress biomarkers, runners should consider proper hydration during the marathon, rehydration post-marathon, and adequate caloric intake with nutrient dense foods and electrolyte replenishment following the race (McDermott et al., 2017). Future research is warranted for sex differences related to renal stress. Further, we did not study factors contributing to, or inhibiting, hydration and renal stress recovery. Future studies are warranted on the mechanistic contributors to renal stress, and how recovery is facilitated so that appropriate advice can be provided for distance runners.

### Limitations

Unfortunately, due to the environmental circumstances that prevented us from collecting blood samples pre-marathon, we were not able to collect pre-marathon_S_Cr or_S_Cys. This limitation prevented us from being able to compare either_S_Cr or_S_Cys values post-marathon to pre-marathon values and determine some level of recovery. We were prevented from providing a clinical diagnosis of AKI determined by increases in_S_Cr (Kellum and Lameire, 2013).

Furthermore, without baseline_S_Cr values, we were unable to calculate an estimate of glomerular filtration rate. However, we were able to determine that neither_S_Cys nor_U_NGAL resolved between post-marathon and 24-h post-marathon, suggesting that renal function was compromised for at least ∼24 h following the Boston Marathon. Compared to immediately post-marathon,_S_Cr did show a significant decrease at 24-h post-marathon suggesting that this biomarker would continue to decrease until recovered. Further research is needed (1) to better understand independent factors or codependence of factors contributing to the differences regarding the Boston Marathon compared to other marathons and (2) data collection at different times post-marathon (48, 72 h, etc.) is warranted to elucidate when short-term biomarkers of renal stress are resolved and (3) to determine if sex differences found in our study are able to be replicated and what mechanisms may contribute to these differences?

## Conclusion

Other studies on marathon runners have demonstrated apparent kidney stress post-marathon but that biomarkers are reduced to near baseline levels 24 h after marathon completion (McCullough et al., 2011; Mansour et al., 2017). Our results differed, suggesting the characteristics unique to the Boston Marathon – increased heat strain, a negative elevation profile in the first 10 miles, and a more elite field of runners resulting in faster finish times – attributed to unresolved renal stress at 24-h post-marathon. Future study related to sex differences in hydration and kidney stress surrounding distance running is warranted.

## Data Availability Statement

The raw data supporting the conclusions of this article will be made available by the authors, without undue reservation.

## Ethics Statement

The studies involving human participants were reviewed and approved by the University of Arkansas Institutional Review Board. The patients/participants provided their written informed consent to participate in this study.

## Author Contributions

WA, CB, MK, DE, BM, and CT conceived and designed the research. WA, CB, AD, RL, and MK collected the data. WA and MR-C analyzed the data. WA, BM, DE, and MK interpreted the results. WA drafted the manuscript. All authors edited, revised, and approved the final version of the manuscript.

## Conflict of Interest

The authors declare that the research was conducted in the absence of any commercial or financial relationships that could be construed as a potential conflict of interest.

## Publisher’s Note

All claims expressed in this article are solely those of the authors and do not necessarily represent those of their affiliated organizations, or those of the publisher, the editors and the reviewers. Any product that may be evaluated in this article, or claim that may be made by its manufacturer, is not guaranteed or endorsed by the publisher.
